# Determination of Common microRNA Biomarker Candidates in Stage IV Melanoma Patients and a Human Melanoma Cell Line: A Potential Anti-Melanoma Agent Screening Model

**DOI:** 10.3390/ijms24119160

**Published:** 2023-05-23

**Authors:** Elena Antonova, Anastasia Hambikova, Denis Shcherbakov, Vitaly Sukhov, Sonya Vysochanskaya, Inna Fadeeva, Denis Gorshenin, Ekaterina Sidorova, Maria Kashutina, Alina Zhdanova, Oleg Mitrokhin, Nadezhda Avvakumova, Yury Zhernov

**Affiliations:** 1 Research Center for Fundamental and Applied Problems of Bioecology and Biotechnology, I.N. Ulyanov Ulyanovsk State Pedagogical University, 432700 Ulyanovsk, Russia; 2 Department of General Hygiene, F. Erismann Institute of Public Health, I.M. Sechenov First Moscow State Medical University (Sechenov University), 119435 Moscow, Russia; 3 Department of English Language, Institute of World Economy, Diplomatic Academy of the Russian Foreign Ministry, 119992 Moscow, Russia; 4 Laboratory of Innate Immunity, National Research Center—Institute of Immunology FMBA of Russia, 115522 Moscow, Russia; 5 Loginov Moscow Clinical Scientific and Practical Center, 111123 Moscow, Russia; 6 Department of Public Health Promotion, National Research Centre for Therapy and Preventive Medicine, 101990 Moscow, Russia; 7 Department of Therapy, Clinical Pharmacology and Emergency Medicine, A.I. Yevdokimov Moscow State University of Medicine and Dentistry, 127473 Moscow, Russia; 8 Department of Medical Chemistry, Samara State Medical University, 443099 Samara, Russia; 9 Department of Chemistry, M.V. Lomonosov Moscow State University, 119991 Moscow, Russia; 10 Center of Life Sciences, Skolkovo Institute of Science and Technology, 121205 Moscow, Russia; 11 Center for Medical Anthropology, N.N. Miklukho-Maclay Institute of Ethnology and Anthropology of the Russian Academy of Sciences, 119017 Moscow, Russia

**Keywords:** melanoma, microRNA, non-coding RNA, plasma biomarkers, exosomes, tumor biomarkers, humic substances, chitosan

## Abstract

MicroRNAs (miRNAs) are small, non-coding RNAs that play an important role in regulating gene expression. Dysregulation of miRNA expression is commonly observed in cancer, and it can contribute to malignant cell growth. Melanoma is the most fatal type of skin malignant neoplasia. Some microRNAs can be prospective biomarkers for melanoma in stage IV (advanced) at higher risk of relapses and require validation for diagnostic purposes. This work aimed to (1) determine the most significant microRNA biomarker candidates in melanoma using content analysis of the scientific literature, (2) to show microRNA biomarker candidates’ diagnostic efficacy between melanoma patients and healthy control groups in a small-scale preliminary study by blood plasma PCR analysis, (3) to determine significant microRNA markers of the MelCher human melanoma cell line, which are also detected in patients with melanoma, that can be used as markers of drug anti-melanoma activity, and (4) test anti-melanoma activity of humic substances and chitosan by their ability to reduce level of marker microRNAs. The content analysis of the scientific literature showed that *hsa-miR-149-3p*, *hsa-miR-150-5p*, *hsa-miR-193a-3p*, *hsa-miR-21-5p*, and *hsa-miR-155-5p* are promising microRNA biomarker candidates for diagnosing melanoma. Estimating microRNA in plasma samples showed that *hsa-miR-150-5p* and *hsa-miR-155-5p* may have a diagnostic value for melanoma in stage IV (advanced). When comparing ΔCt *hsa-miR-150-5p* and ΔCt *hsa-miR-155-5p* levels in melanoma patients and healthy donors, statistically significant differences were found (*p* = 0.001 and *p* = 0.001 respectively). Rates ΔCt were significantly higher among melanoma patients (medians concerning the reference gene *miR-320a* were 1.63 (1.435; 2.975) and 6.345 (4.45; 6.98), respectively). Therefore, they persist only in plasma from the melanoma patients group but not in the healthy donors group. In human wild-type stage IV melanoma (MelCher) cell culture, the presence of *hsa-miR-150-5p* and *hsa-miR-155-5p* in supernatant was detected. The ability of humic substance fractions and chitosan to reduce levels of *hsa-miR-150-5p* and *hsa-miR-155-5p* was tested on MelCher cultures, which is associated with anti-melanoma activity. It was found that the hymatomelanic acid (HMA) fraction and its subfraction UPLC-HMA statistically significantly reduced the expression of *miR-150-5p* and *miR-155-5p* (*p* ≤ 0.05). For the humic acid (HA) fraction, this activity was determined only to reduce *miR-155-5p* (*p* ≤ 0.05). Ability to reduce *miR-150-5p* and *miR-155-5p* expression on MelCher cultures was not determined for chitosan fractions with a molecular weight of 10 kDa, 120 kDa, or 500 kDa. Anti-melanoma activity was also determined in the MTT test on MelCher cultures for explored substances. The median toxic concentration (TC50) was determined for HA, HMA and UPLC-HMA (39.3, 39.7 and 52.0 μg/mL, respectively). For 10 kDa, 120 kDa, or 500 kDa chitosan fractions TC50 was much higher compared to humic substances (508.9, 6615.9, 11352.3 μg/mL, respectively). Thus, our pilot study identified significant microRNAs for testing the in vitro anti-melanoma activity of promising drugs and melanoma diagnostics in patients. Using human melanoma cell cultures gives opportunities to test new drugs on a culture that has a microRNA profile similar to that of patients with melanoma, unlike, for example, murine melanoma cell cultures. It is necessary to conduct further studies with a large number of volunteers, which will make it possible to correlate the profile of individual microRNAs with specific patient data, including the correlation of the microRNA profile with the stage of melanoma.

## 1. Introduction

Melanoma is one of the most fatal kinds of skin neoplasms [1,2]. The importance of the early diagnosis of melanoma is determined by the high aggressiveness of the disease development, early metastasis of the tumor, the rising incidence of melanoma among young and middle-aged working-age people, and the lack of satisfactory treatment results [3,4,5]. Early-stage diagnostics of melanoma gives the opportunity for tumor surgical resection, which is an appropriate therapeutic approach, greatly enhancing the survival of the patient. On the contrary, surgical treatment alone does not sufficiently improve patient outcomes in advanced metastatic stages of melanoma [6]. Primary melanoma patients have an 11% mortality rate, whereas the mortality due to metastatic melanoma is significantly higher [7]. The median survival time after distant metastasis onset is only 6–9 months, and the 5-year survival rate is less than 5% [1]. Therefore, better treatment of this malignancy requires the development of newer approaches to diagnose melanoma in its early stages and treat it in its advanced stages. Considering this need, data on molecular alterations in melanoma are extremely valuable for the discovery of novel therapeutic targets and biomarkers that allow for early melanoma detection.

MicroRNAs, a subtype of non-coding RNA, are a well-recognized gene expression regulator in mammalian cells and play a significant role in malignant cell growth when dysregulated [8]. They bind to response elements in the 3’ untranslated region (3’-UTR) of messenger RNA (mRNA) molecules, leading to post-transcriptional silencing of gene expression by degradation or translational repression of target mRNAs [9].

The role of microRNA as one of the main causative factors in malignant cell growth was first suggested in 2002 [10]. In chronic lymphocytic leukemia (CLL), B-cell expression of microRNAs (*miR15* and *miR16*), which are required for normal CD5+ B-cell differentiation, was significantly downregulated or absent due to 13q14 locus allelic loss, which was observed in 68% of all CLL cases [10]. Today, there is an extensively growing amount of data about microRNA dysregulation in the transcriptomes of different cancer types. MicroRNA expression profiles changed during the development of most malignant tumors, suggesting that microRNAs can act as oncogenes, tumor suppressors, and drivers of malignant transformation [11]. There is also increasing evidence of microRNA dysregulation in melanoma [12].

According to studies, cells can release certain types of microRNAs into the extracellular space [8]. To date, numerous data on extracellular or circulating microRNA have been obtained in various biological fluids (e.g., plasma, serum, cerebrospinal fluid, saliva, breast milk, urine, tears, colostrum, peritoneal fluid, bronchial lavage, seminal fluid, and ovarian follicular fluid) [8]. Some of these microRNAs can be utilized as biomarkers for a wide spectrum of diseases, including different cancer types (e.g., melanoma). Extracellular microRNAs, in contrast to cellular RNA species, are relatively stable and resistant to degradation at room temperature for up to 4 days and in adverse conditions such as boiling, multiple freeze-thaw cycles, and high or low pH [13,14], making circulating microRNA a convenient target for various diagnostic applications. Validation of these microRNAs as melanoma biomarkers for clinical usage may provide a relatively simple and inexpensive method for melanoma diagnostics that may also be potentially applied for cancer screening or dynamic monitoring of the disease progression and treatment efficacy.

In recent years, many microRNAs have been shown to have potential clinical relevance for melanoma diagnostics, prognosis, and treatment outcomes [1,6,7]. Some of these microRNAs, such as *miR-214*, are shown to be pleiotropic, i.e., they are deregulated in several other tumors besides melanoma and other skin cancers [15]. Some others are specific to one or more skin cancer types, like *miR-21* and *miR-221* for cutaneous melanoma and cutaneous squamous carcinoma, or *miR-155* for melanoma and cutaneous lymphoma [15].

One of the first discovered circulating microRNAs that is relevant to melanoma was *miR-221*. *MiR-221* is aberrantly expressed in melanoma cells and can also be detected in the serum of patients diagnosed with cutaneous melanoma [15]. It is known that *miR-221* downregulates expression of some genes with tumor-suppressing function in melanoma, e.g., cyclin-dependent kinase inhibitor 1B (CDKN1B/p27Kip1), and c-KIT receptor (CD117) [16]. Quantitative real-time polymerase chain reaction (qRT-PCR) has shown that *miR-221* levels are significantly higher in melanoma patients’ serum compared to controls. Moreover, the serum level correlated with stage, the tumor thickness, recurrence, and worse outcome, which shows *miR-221* as a good example of a microRNA biomarker in melanoma [17,18]. Therefore, some extracellular tumor-specific microRNAs can be used as biomarkers to expand diagnostic information [19]. For instance, some microRNAs also determine melanoma resistance to different therapies [20].

Moreover, microRNAs, which are detected in patient plasma, can be used as markers of promising drugs anti-melanoma activity. It is known that microRNAs in tumors can be a target for the antitumor action of various drugs, since microRNAs play an important role in initiating tumor growth [21]. Decrease of such microRNA levels can be assayed in vitro using human cell culture models, for example MelCher human melanoma cell line.

It is noted that various research literature sources often provide scattered information on the levels of microRNA expression in blood and tissues with melanoma and different assumptions about their role in the diagnosis of disease [8]. While studying microRNAs as melanoma biomarkers, we find it essential to consider the profiles of several types of biomarkers simultaneously. The main problem in melanoma is to follow-up patients with melanoma in stage IV (advanced) at higher risk of relapses. This is possible by the detection of melanoma specific somatic mutations in circulating cell-free DNA (ccfDNA) from plasma samples [22]. Nevertheless, this approach is not possible for patients with wild type melanomas. In the latter case, microRNA, can be candidate biomarkers.

Our work aimed to (1) determine the most significant microRNA biomarker candidates in melanoma using content analysis of the scientific literature, (2) to show microRNA biomarker candidates’ diagnostic efficacy between melanoma patients and healthy control groups in a small-scale preliminary study by blood plasma PCR analysis, (3) to determine significant microRNA markers of the MelCher human melanoma cell line, which are also detected in patients with melanoma, that can be used as markers of drug anti-melanoma activity, and (4) test anti-melanoma activity of humic substances and chitosan by their ability to reduce level of marker microRNAs.

## 2. Results

### 2.1. Determine of microRNA Biomarker Candidates

The conducted content analysis of the scientific literature allowed us to form a list of microRNAs expressed differently in melanoma patients compared to healthy donors (Table 1).

In addition, we have compiled a list of microRNAs that are expressed differently in patients with metastatic melanoma compared with patients with non-metastatic melanoma and/or those whose up-or down-regulation correlates with a poor prognosis of the disease (Table 2).

Based on the content analysis results, we have selected five microRNAs overexpressed during tumor growth that we accept are candidates for melanoma biomarkers: *hsa-miR-149-3p*; *hsa-miR-150-5p*; *hsa-miR-193a-3p*, *hsa-miR-21-5p*, and *hsa-miR-155-5p*. For instance *miR-150-5p*, *hsa-miR-21-5p*, *hsa-miR-155-5p* and *miR-149-3p* appear only once in the results of content analysis in the Table 2. In comparison, other genes, such as *miR-146a*, are reproduced by more than one study. *miR-193a-3p* is a down-regulated circulating miRNA and would be an candidate when a close sequence *miR-193b-3p* appears in content analysis twice as an overexpressed miRNA.

At the same time, these five microRNA biomarkers may have diagnostic value. *Hsa-miR-149-3p* plays a critical role in the process of cell migration [26,40]. *MiR-193a-3p* linked to BRAF mutation status in melanoma tissues [36,41]. *Hsa-miR-150-5p* plays an essential role in hematopoiesis, regulating genes that reduce the expression level of products involved in the differentiation of stem cells [42,43]. *Hsa-miR-155-5p* plays an essential role in various physiological and pathological processes. It takes part in suppressing viral infections and the growth of malignant tumors, etc. It is valuable that this type of microRNA can be transmitted through exosomes [29]. Released tumor exosomal microRNAs play a crucial role in programming its microenvironment. It was also previously noted that exosomal *hsa-miR-155-5p* is involved in controlling angiogenesis in melanoma [44]. *Hsa-miR-21-5p* regulates some cancer-related gene expressions, s.a. *PTEN*, *TIMP3*, *RHOBPTEN*, *COAD*, *PDCD4*, and *BTG2*, and is associated with various pathological processes, incl. oncogenesis [45,46,47,48]. A high level of *hsa-miR-21-5p* expression is a negative predictor of survival in multiple forms of cancer. This type of microRNA can be found in plasma and other extracellular fluids [32].

### 2.2. Pilot Study Results

Based on the content analysis results, we further identified five microRNA in plasma samples from melanoma patients and healthy donors: *hsa-miR-149-3p*, *hsa-miR-150-5p*, *hsa-miR-193a-3p*, *hsa-miR-21-5p*, and *hsa-miR-155-5p*. The miRBase database (www.mirbase.org, accessed on 20 March 2023) was used to obtain sequence data for these five candidate diagnostic microRNAs (Table 3).

**Figure 1:**
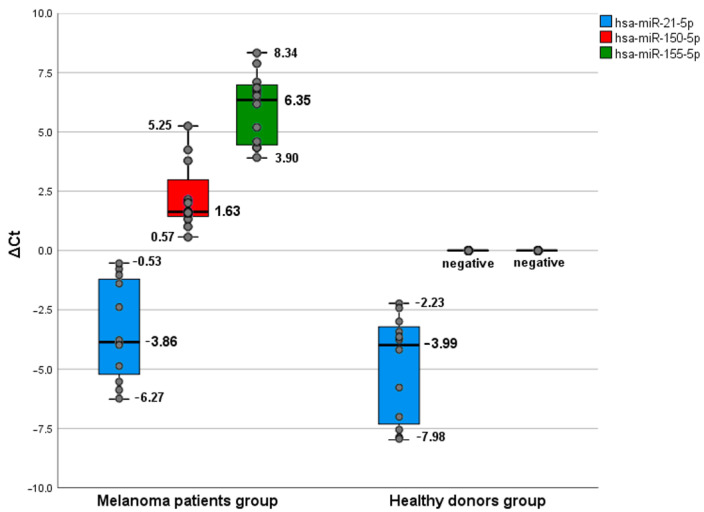
The boxplot of levels of serum *hsa-miR-150-5p*, *hsa-miR-21-5p*, and *hsa-miR-155-5p*. There was a difference between the stage IV melanoma patient (*n* = 12) and the healthy donor (*n* = 12) serum microRNAs to distinguish using qRT-PCR. Lower and upper box boundaries represent the 25th and 75th percentiles, respectively; the line inside the box represents the median; and the lower and upper error lines represent the min and max percentiles, respectively. All promising microRNA biomarker candidates were normalized with reference gene *miR-320a*.

The qRT-PCR analysis obtained in the stage IV melanoma patients group identified 3 out of 5 studied microRNAs: *hsa-miR-21-5p*, *hsa-miR-150-5p*, *hsa-miR-155-5p*. The lack of detection of *hsa-miR-149-3p* and *hsa-miR-193a-3p* can be explained by their low concentration (which makes them undetectable by the test system) or their complete absence in plasma samples (Figure 1). When comparing ΔCt *hsa-miR-150-5p* and ΔCt *hsa-miR-155-5p* levels in melanoma patients and healthy donors, statistically significant differences were found (*p* < 0.001 and *p* < 0.001 respectively). Rates ΔCt were significantly higher among melanoma patients (medians concerning the reference gene *miR-320a* were 1.63 (1.435; 2.975) and 6.345 (4.45; 6.98), respectively).

**Figure 2:**
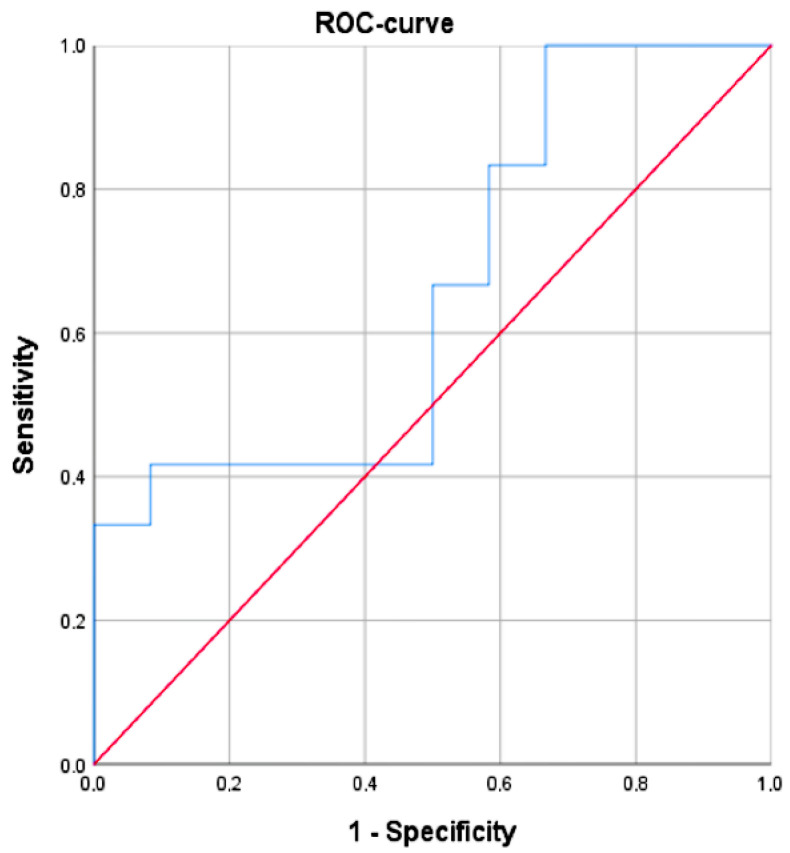
The ROC-curve characterizes the dependence of the probability of diagnosing stage IV melanoma in a patient depending on the ΔCt *hsa-miR-21-5p* level.

Differences in ΔCt *hsa-miR-21-5p* levels depending on the presence of melanoma were not statistically significant (*p* = 0.21). The area under the ROC curve corresponding to the relationship between the detection of melanoma in a patient and the level (delta) of hsa-miR-21-5p was 0.714 ± 0.143 with 95% CI: 0.433–0.996 (Figure 2). The resulting model was statistically insignificant (*p* = 0.18). Thus, the use of *hsa-miR-21-5p* in the diagnosis of melanoma is impossible according to our estimates.

**Figure 3:**
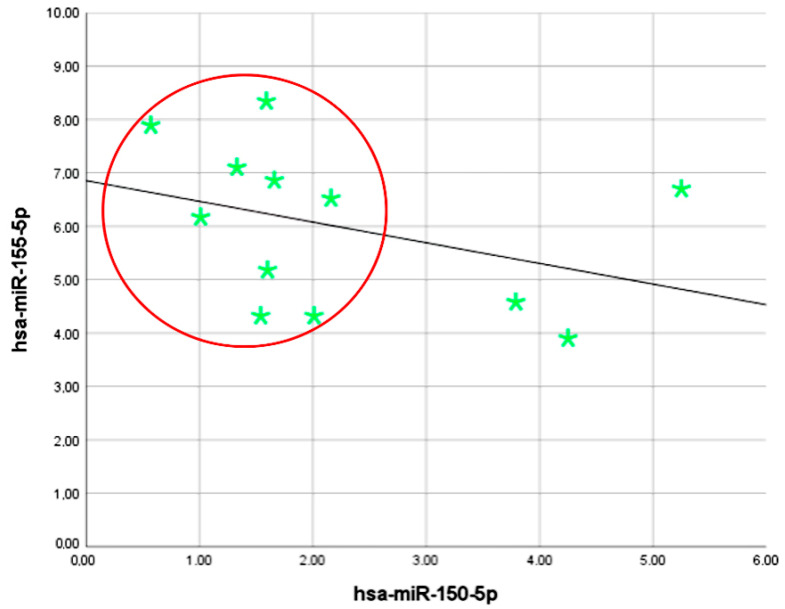
The scatter plot of the combination of Ct values for both *hsa-miR-150-5p* and *hsa-miR-155-5p*. The individual Ct values of each stage IV melanoma patient are represented by green stars; the trend line is represented by the black line; and clustering is represented by the red circle.

As a result of the correlation analysis of the ΔCt *hsa-miR-150-5p* level and the ΔCt *hsa-miR-155-5p* level, a statistically significant direct correlation of strong closeness was established (ρ = 0.798; *p* < 0.001). An increase in hsa-miR-150-5p levels among patients diagnosed/confirmed with melanoma was accompanied by higher hsa-miR-155-5p values. According to the Chadock’s scale, the revealed relationship had high tightness. Scatter plotting of normalized Ct values for both hsa-miR-150-5p and hsa-miR-155-5p shows the unique cluster of melanoma including 9 of the 12 patients (Figure 3). It is conceivable that the combined measurement of both microRNAs could increase the diagnostic value in melanoma patients.

### 2.3. Potential Anti-Melanoma Agent Screening Model Based on Human Melanoma Cell Line

**Figure 4:**
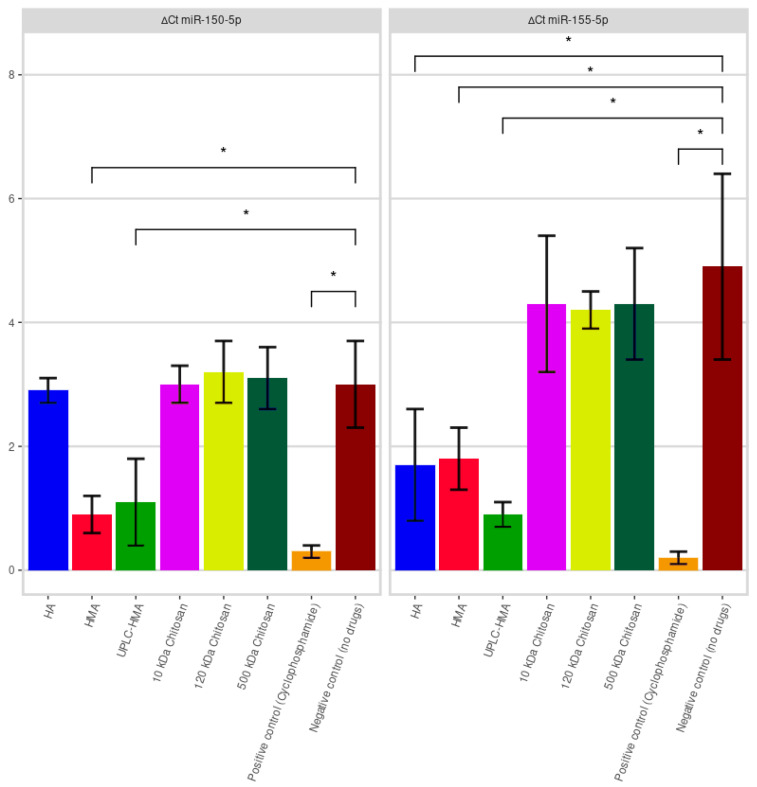
Expression level of *miR-150-5p* and *miR-155-5p* of human stage IV melanoma cell line MelCher in the supernatant exposed to fractions of humic substances, chitosans (10 kDa, 120 kDa, and 500 kDa) and cyclophosphamide; *n* = 3, * *p* ≤ 0.05 from *miR-150-5p* and *miR-155-5p* negative control.

In order to test the anti-melanoma activity of humic substance fractions or chitosan by reducing the expression (suppression) of microRNA in melanoma cells, we conducted an in vitro study with the MelCher culture. *MiR-150-5p* and *miR-155-5p*, identified as promising miRNAs in a pilot patient study, were detected in collected supernatants by qRT-PCR. The concentrations of fractions of humic substances and chitosan were 100 μg/mL. The positive control was the cytostatic agent Cyclophosphamide at a concentration of 100 μg/mL. Supernatants were collected after the MelCher cells without the addition of drugs used as a positive control. The negative control was the culture medium without melanoma cells. The results of the study are presented in Figure 4.

The mean ∆Ct for *miR-150-5p* and *miR-155-5p* in the supernatant of MelCher cell line exposed to fractions of humic substances (HA, HMA, and UPLC-HMA), chitosan (10 kDa, 120 kDa, and 500 kDa), positive control with cyclophosphamide and negative control without drugs (M ± SD; *n* = 3) were, respectively, 2.9 ± 0.2, 0.9 ± 0.3, 1.1 ± 0.7, 3.0 ± 0.3, 3.2 ± 0.5, 3.1 ± 0.5, 0.3 ± 0.1, 3.0 ± 0.7 for *miR-150-5p*, and 1.7 ± 0.9, 1.8 ± 0.5, 0.9 ± 0.2, 4.3 ± 1.1, 4.2 ± 0.3, 4.3 ± 0.9, 0.2 ± 0.1, 4.9 ± 1.5 for *miR-155-5p*.

It was found that the HMA fraction and its subfraction UPLC-HMA statistically significantly reduced the expression of *miR-150-5p* and *miR-155-5p* in MelCher human stage IV melanoma cell culture. For the HA fraction, this activity was determined only to reduce *miR-155-5p* compared to the control. Anti-melanoma activity was not determined for chitosan fractions with a molecular weight of 10 kDa, 120 kDa, or 500 kDa in this cell model.

### 2.4. Cytotoxicity Assay in MTT Test

**Figure 5:**
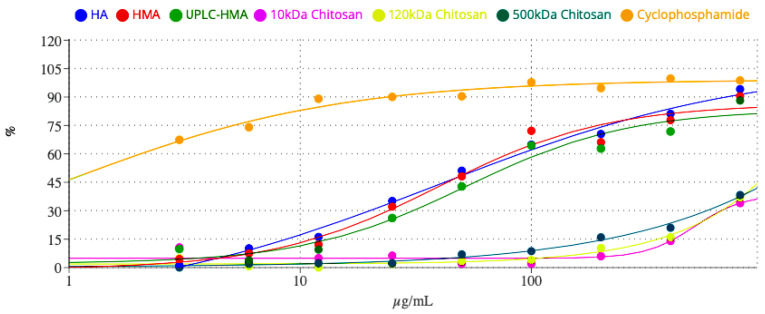
Dose-response curves of humic substances and chitosans fractions in MelCher human melanoma cell culture.

Dose-response curves of cytotoxicity in the MTT test were built for assessed preparations. The curves are given in Figure 5.

**Figure 6:**
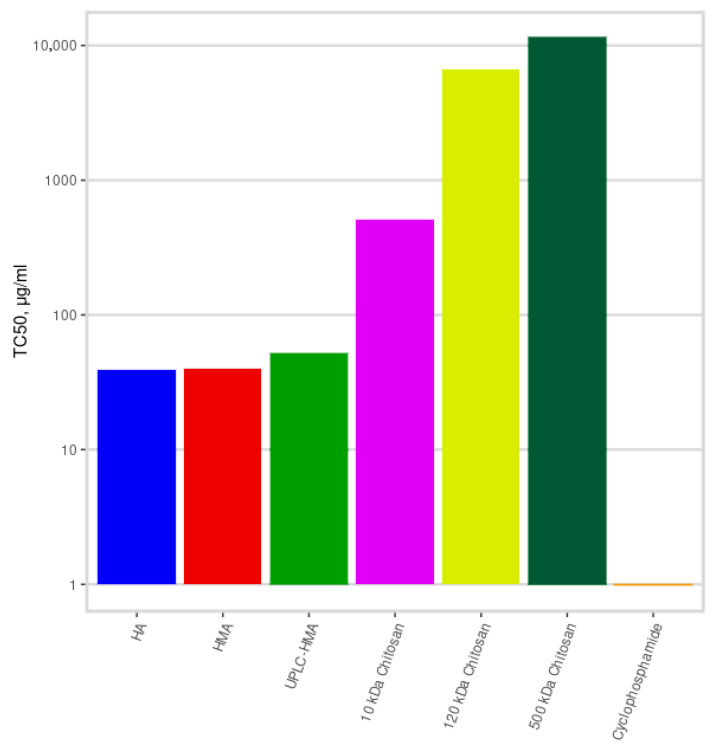
Median toxic concentration (TC50) of fractions of humic substances and chitosans on MelCher human melanoma cell culture, Y axis is in log_10_ scale.

The median toxic concentrations (TC50) for examined drugs were calculated and presented in Figure 6.

The direct cytotoxic activity of the chitosan fractions turned out to be low at a level of 508.9, 6615.9, 11,352.3 μg/mL, while with an increase in the molecular weight, the anti-melanoma activity of the fractions decreased.

The maximum anti-melanoma activity on MelCher cells was recorded in the HA fraction (TC50 = 39.3 μg/mL). HMA fraction showed almost the same activity (TC50 = 39.7 μg/mL). UPLC-HMA fraction showed slightly lower activity (TC50 = 52.0 μg/mL).

## 3. Discussion

As can be seen, microRNAs represent promising potential diagnostic and prognostic melanoma biomarkers. Our research and content analysis showed limited agreement between circulating microRNA panels identified by different research groups. So, we have only a few potential and clinically valid predictive, prognostic, and diagnostic microRNAs for melanoma. The most reliable potential biomarkers among published circulating microRNAs were identified in several studies belonging to the same research direction and further preferentially verified with an independent validation cohort using an acceptable normalization method.

Our analysis showed that, among all the studied promising microRNAs, *hsa-miR-150-5p* and *hsa-miR-155-5p* are of diagnostic value for melanoma in stage IV (advanced). These microRNAs were not detected in the control group plasma of healthy donors, while their median concentration concerning the reference gene *miR-320a* reached 2.74 (1.625; 2.795) and 5.78 (4.67; 7.4), respectively. Moreover, the use of a combination of these two microRNA markers may provide more predictive potential for the diagnosis of melanoma. *Hsa-miR-149-3p*, *hsa-miR-193a-3p*, and *hsa-miR-21-5p* showed low informative value in melanoma diagnostics demonstrated by our studies.

As the test revealed, both groups, the healthy volunteers’ and the melanoma patients’ groups, had *hsa-miR-21-5*, 1 out of 5 studied microRNAs. The median ∆Ct of this microRNA was −4.42 in the control group and −3.67 in melanoma patients (Figure 2). A wide range of values in both groups does not consider this type of microRNA as informative and is not statistically significant (*p* = 0.21). The differences in this microRNA levels among patients, including the experimental subjects in the group, were probably connected with other factors. The lack of detection of *hsa-miR-149-3p* and *hsa-miR-193a-3p* could be explained by their low levels (which makes them undetectable by the test system) or their complete absence in plasma samples. At the same time, *hsa-miR-150-5p* and *hsa-miR-155-5p* were detected in much higher levels in melanoma patients than healthy donors.

Diagnostic microRNA biomarkers have certain advantages. Due to its structure, the microRNA molecules are relatively stable, which allows it to be isolated from all biological fluids, including after freezing (scraping from the cheek, saliva, plasma, blood, urine, etc.). Moreover, microRNA molecules can be contained in two forms as free microRNAs and in exosomes. For both cases, there are standard research protocols. However, due to specificity problems and some difficulties of the analytical stage (the issue of normalizing the obtained PCR data), the desire to use microRNA as a primary diagnostic tool has recently decreased. Still, at the same time, other, more realistic, in our opinion, prospects are opening up for microRNAs: monitoring of ongoing therapy and use as therapeutic molecules. Advantages of these microRNA as markers of anti-melanoma activity for in vitro assays are also pronounced. In order to test the anti-melanoma activity of humic substance fractions and chitosan by reducing the expression of microRNA in melanoma cells, we conducted an in vitro study with the MelCher culture. *miR-150-5p* and *miR-155-5p* were detected in collected supernatants by qRT-PCR. It was found that the HMA fraction and its subfraction UPLC-HMA statistically significantly reduced the expression of *miR-150-5p* and *miR-155-5p* in MelCher human melanoma cell culture. For the HA fraction, this activity was determined only to reduce *miR-155-5p* compared to the control. Anti-melanoma activity on MelCher cultures was not determined for chitosan fractions with a molecular weight of 10 kDa, 120 kDa, or 500 kDa. Despite the anticancer activity described in the sources [49,50,51,52,53,54,55], chitosans did not show the ability to reduce *miR-150-5p* and *miR-155-5p* expression in the MelCher cell model. However, further studies are needed to study anti-melanoma activity of chitosans on other melanoma cell cultures.

Such a cell model can be used to test the anti-melanoma activity of other potential active substances. Using human melanoma cell cultures gives opportunities to test new drugs on the culture that has a microRNA profile similar to patients with melanoma unlike, for example, murine melanoma cell cultures.

In clinical practice, it is hard to assess cytotoxicity of prospective drugs, and microRNA markers give opportunity to check the effectivity of treatment, because TC50 correlated with the results of the miRNA assay with 0.86 (*p* = 0.024) for *miR-150-5p* and 0.83 (*p* = 0.021) for *miR-155-5p*.

## 4. Materials and Methods

### 4.1. Study Design

This small-scale preliminary (pilot) study was divided into six steps: (1) content analysis of the scientific literature to determine the underutilized biomarkers among microRNAs in melanoma; (2) patient characterization and blood sampling for further investigation. The pilot study groups included 12 responders per group [56]: 5 healthy male and 7 healthy female donors with a mean age of 53,3 years and 5 male and 7 female melanoma patients with a mean age of 54.8 years. The melanoma patients were all in stage IV (advanced); (3) blood sample preparation. Steps (4) and (5) were the last two steps, which comprised microRNA sample isolation and qRT-PCR analysis, and calculation of results and statistical analysis.

The findings of this pilot study on a small number of subjects allowed implementation of the obtained data on promising microRNA to create a potential anti-melanoma agent screening model (Step 6) based on a wild-type human melanoma (MelCher) cell line. This model was tested with a cytostatic agent Cyclophosphamide (positive control), as well as promising natural preparations of fractions of humic substances and chitosan with high biological activity.

### 4.2. Content Analysis of the Scientific Literature

To determine the most promising biomarkers among microRNAs in melanoma, we carried out a content analysis of the scientific literature on this topic over the past 15 years, using the PubMed database for the subsequent practical pilot study. Preference was given to original research. Key words used were: ‘melanoma’, ‘stage IV’, ‘microRNA’, ‘non-coding RNA’, ‘circulating microRNA’, ‘plasma biomarkers’, ‘exosomes’, ‘tumor biomarkers’. MicroRNAs for our study were selected based on the high frequency of mention in selected articles.

### 4.3. Patient Characteristics and Blood Collection

Blood samples were collected from melanoma patients (12 people) and healthy donors (12 people) at the Ulyanovsk Regional Oncological Clinic (Ulyanovsk, Russia) and the N.N. Petrov National Medicine Research Center of oncology (St. Petersburg, Russia). In terms of the number of subjects and stage of melanoma, the pilot study groups were homogeneous. All study subjects signed informed consent. The characteristics of responders by gender, age, and diagnosis are presented in Table 4.

### 4.4. Blood Sample Preparation

Blood sampling (arr. 1 mL) was carried out in vacuum tubes with EDTA. Immediately after blood sampling, the samples were centrifuged at 4 °C (39.2 °F) for 10 min at 1900× *g*. Then plasma was collected in sterile 1.5 mL Eppendorf tubes and stored at −80 °C (−112 °F).

### 4.5. Isolation of microRNA Samples and qRT-PCR Analysis

One qRT-PCR analysis required 200 μL of blood plasma. The isolation of microRNAs from blood plasma was achieved by mirVana™ miRNA Isolation Kit (Ambion/Thermo Scientific, Waltham, MA, USA). As the exogenous control, we used synthetic microRNA, cel-miR-39-3p, in 2 μL of 0.05 μM solution per 200 μL of plasma. The reverse transcription was carried out with TaqMan Advanced miRNA cDNA Synthesis Kit (Thermo Scientific, Waltham, MA, USA). RNA samples were stored at −80 °C (−112 °F).

The real-time PCR (qRT-PCR) was performed using TaqMan Fast Advanced Master Mix (Thermo Scientific, Waltham, MA, USA), TaqMan™ Advanced miRNA Assay (Thermo Scientific, Waltham, MA, USA) *hsa-miR-21–5p*, *hsa-miR-149–3p*, *hsa-miR-150–5p*, *hsa-miR-155–5p*, *hsa-miR-193a-3p*.

### 4.6. Cell Lines and Cell Culture

Wild-type human melanoma (MelCher) cell line was used for experiments. A wild-type human melanoma cell line was obtained from a patient with cutaneous melanoma (stage IV) [57]. Cells were stored at liquid nitrogen temperature. Cells were cultured in RPMI-1640 medium containing 10% fetal bovine serum (FBS), 2 mM L-glutamine, 40 μg/mL gentamicin at 37 °C in an atmosphere of 5% CO_2_. The cells were passaged on the third day. The cell monolayer was removed with Hank’s solution (without Ca^2+^ and Mg^2+^) containing 0.05% trypsin and 0.53 mM EDTA at 37 °C. The effect of trypsin was neutralized by adding 10% FBS to RPMI-1640.

### 4.7. Obtaining Fractions of Humic Substances and Chitosan

Melanoma refers to immunoactive tumors, so there is a possibility of studying its treatment through the use of immunomodulatory drugs [58]. One of the promising directions in the search and development of antitumor immunoactive drugs is the use of natural raw materials. The advantage of drugs based on natural substances is the availability of raw materials, the environmental friendliness of its production, a favorable biosafety profile, as well as a relatively low cost compared to synthetic drugs [59]. Many authors have suggested the presence of active natural substances in humic tree fungi-saprotrophs, such as straight-legged melanoleuca (*Melanoleuca strictipes*), birch tinder fungus (*Piptoporus betulinus*), multi-colored trametes (*Coriolus versicolor*), etc. [60,61]. Therefore, in our work, the most common chemical components of such fungi were chosen as candidate anti-melanoma drugs: humic substances, as decomposition products of lignin, a substrate of saprotrophs, as well as chitosans and chitins, components of the cell wall of such fungi.

Humic substances are heteropolymers of natural origin [62], which are already used in clinical practice as a pharmacopoeial enterosorbent preparation based on hydrolytic lignins (*Ligninum hydrolisatum*) in the form of Polyphepan, Filtrum STI and Polyfan [63]. There are suggestions that humic substances have a mechanism of immunostimulating activity due to the presence of organic nitrogen, which is capable of inducing cytokines and activating cells of the immune system [64]. This group of substances has a previously described broad biological activity [64,65,66,67,68,69], which makes it promising for testing in a potential anti-melanoma agent screening model. There is evidence that lignins can influence the activation of the immune response in vitro. Oxyhumate (a water-soluble hydrolysis lignin derived from coal) has been shown to increase Th-1 cell activity while decreasing Th-2 cytokine production [70]. The observed stimulation of the proliferation of phytohemagglutinin-stimulated human lymphocytes was associated with an increase in the production of interleukin-2 (IL2) and the expression of IL2 receptors, together with a decrease in the amount of interleukin-10 (IL10) under the action of Oxyhumate, the concentration of which in the experiment ranged from 20 μg/ml and above [71]. In another in vivo study, it was shown that oral administration of hydrolytic lignins improves the parameters of innate immunity in experimental animals: there is an increase in the antibacterial activity of blood serum, phagocytic activity, lysozyme activity, and bacterial agglutination [72].

We obtained fractions of humic substances: the alkaline-soluble fraction of humic acid (HA), the alcohol-soluble fraction of hymatomelanic acid (HMA) and its ultra-high performance liquid chromatography subfraction (UPLC-HMA). The extraction procedure of humic substances is described in Supplementary Materials (File S1).

Chitosan is a biopolymer, in most cases obtained by a semi-synthetic method during the processing of chitin, and consisting of β-(1-4)-D-glucosamine units and N-acetyl-D-glucosamine. Chitosan meets a number of the most important requirements for pharmaceutical raw materials: biocompatibility with body tissues, a high safety profile and, most importantly, ample opportunities for modification and application [49,50]. Modified water-soluble chitosan oligomers are able to maintain stability at pH values close to physiological and, subject to the rules of administration and the preservation of molecular structure, their use does not lead to the development of embolism. A number of publications in the scientific literature are devoted to the direct effect of chitosan derivatives on tumor growth both in experiments in vivo and in cell cultures [51]. One of the possible mechanisms of the chitosan direct effect on tumor cells is adhesion on their surface due to the pronounced positive charge of amino groups and the relatively negatively charged tumor cell compared to normal cells, which leads to difficulty in intercellular interactions and inhibition of tumor growth [52]. Another possible mechanism is inhibition of the activity of matrix metalloproteinase-9 (MMP-9), an enzyme associated with the activity of endothelial growth factor (VEGF). Accordingly, inhibition of MMP-9 leads to a decrease in VEGF expression, which leads to the degradation of the tumor structure [53]. In experiments on various cell lines (BGC-823, SGC-7901, A549, NCI-H460, KCC-853, 786-O, HCT-116, HT-29 and MCF-7), the antitumor effect of chitooligomers was shown in a wide range of dosages [54]. In another work, in vitro experiments showed a high antitumor activity of chitosan derivatives on the PA-1 cell line. At a concentration of 10 µg/mL, almost complete cessation of tumor cell growth was achieved [55].

In our work, we used three fractions of chitosan with a molecular weight of 10 kDa, 120 kDa, and 500 kDa, produced by Bioprogress Ltd., Shchyolkovo (Russia). The procedure of obtaining chitosans is described in Supplementary Materials (File S1) [73].

Thus, the above biological properties of the fractions of humic substances and chitosan make them promising substances for the development of candidate immunomodulatory drugs against melanoma.

### 4.8. Incubation with Candidate Preparations of Humic Substances and Chitosan

To study the anti-melanoma activity of candidate substances in vitro, a human melanoma cell line, previously grown in a 96-well plate, was incubated together with humic substances or chitosan promisings at a concentration of 100 μg/mL. The negative control for anti-melanoma activity was live human melanoma cells cultured only in DMEM nutrient medium without the addition of other tested drugs. The positive control was the cytostatic agent Cyclophosphamide at a concentration of 100 μg/mL. After 24 h of incubation of melanoma cells with candidate drugs, the nutrient medium was replaced (washed). Next, the cells were incubated for 24 h in a new medium without the drug, followed by the collection of the supernatant for the detection of microRNAs in it by the same method as microRNA detection in blood plasma.

### 4.9. Cytotoxicity Assay in MTT Test

The direct cytotoxic effect of the studied fractions of humic substances and chitosans was evaluated using the MTT test. This test is based on the ability of mitochondrial reductases in living melanoma cells to convert and reduce colorless, water-soluble 3-(4,5-dimethylthiazol-2-yl)-2, 5-diphenyltetrazolium bromide (MTT) to violet-blue 3-(4, 5-dimethylthiazol-2-yl)-2,5-diphenyl-2H-formazan.

To do this, 20 μL of MTT solution at a concentration of 8 μg/mL is added to the wells of a 96-well plate with MelCher melanoma cells, after removal of the supercellular fluid for microRNA diagnostics, and left for 4 h in a CO_2_ incubator. Next, the environment is removed. 200 μL of DMSO are added to the wells, the contents are mixed and incubated for 5 min. Measurement of optical density with an ELISA reader at 630 nm made it possible to detect viable MelCher melanoma cells. Based on the results of the experiment, the concentration of the drug TC50 was calculated, at which 50% death of the studied MelCher melanoma cell culture is observed.

### 4.10. Calculation of Results and Statistical Analysis

Each test was carried out in triplicate to calculate the average value. All samples set the threshold Ct value as 0.1 (qPCRsoft v. 3.0 by Analytik Jena AG, Jena, Germany). The calculation of the relative level was carried out using the ΔΔCt method [13]. All promising microRNA biomarker candidates were normalized with reference gene *miR-320a*, chosen by applying the NormFinder algorithm [74].

To assess the normality of the distribution of the analyzed quantitative variables, the Shapiro-Wilk test was used. The analyzed variables had a distribution different from normal, which is why the median (Me) and interquartile range (Q1;Q3) indicators were used in their description, and the Mann-Whitney test was used in the comparative analysis. The Spearman’s rank correlation coefficient (ρ) was used to identify and evaluate relationships between quantitative variables. The correlation was considered statistically significant at *p* < 0.05. The direction of the correlation was regarded as direct at ρ ˃ 0 and reversed at ρ < 0. The tightness of the connection was recognized as weak if ρ < 0.3, medium—at 0.3 ≤ ρ < 0.7, strong—at 0.7 ≤ ρ. The assessment of the characteristics of the closeness of correlation links was carried out using the Chaddock scale. Analysis of ROC-curves with assessment of the area under the curve (AUC) was used to assess the diagnostic significance (predictive value) of microRNAs in patients. The critical level in the work was the value of *p* < 0.05. The International Business Machines Statistical Package for the Social Sciences v.26.0 software (developed by IBM Statistics, Armonk, NY, USA) was used to do the statistical analysis.

## 5. Conclusions

The content analysis of the scientific literature showed that *hsa-miR-149-3p*, *hsa-miR-150-5p*, *hsa-miR-193a-3p*, *hsa-miR-21-5p*, and *hsa-miR-155-5p* are promising microRNA biomarker candidates for diagnosing melanoma.

The assay for *hsa-miR-150-5p* and *hsa-miR-155-5p* in the MelCher cell line established that these microRNAs were present in the supernatant, and tests with humic substance fractions have shown the ability of HMA and UPLC-HMA to suppress levels of these biomarker microRNAs. HA suppressed only *hsa-miR-155-5p* expression. In addition to humic substances, chitosan fractions with a molecular weight of 10 kDa, 120 kDa, or 500 kDa were studied, but they did not show the ability to reduce the *hsa-miR-150-5p* and *hsa-miR-155-5p* expression in the current melanoma model.

Estimating microRNA in plasma samples showed that *hsa-miR-150-5p* and *hsa-miR-155-5p* may have a diagnostic value for melanoma in stage IV (advanced). Therefore, they persist only in plasma from the melanoma patients’ group but not in the healthy donors’ group. *hsa-miR-21-5* was detected in both groups, but a wide range of values in both groups does not consider this microRNA an informative biomarker for melanoma.

It is necessary to conduct further studies with a large number of volunteers, which will make it possible to correlate the profile of individual microRNAs with specific patient data, including to reveal the correlation of the microRNA profile with the stage of melanoma. It is promising to include additional microRNAs in the list of studied ones and increase the sensitivity of the test system used.

## Figures and Tables

**Table 1:** Diagnostic biomarkers of circulating microRNAs for melanoma. Melanoma patients compared with the healthy volunteers.

Overexpressing microRNA-Candidates	Down-Expressing microRNA-Candidates	Sample, Tissue	Source Link
*miR-186*, *let-7d*, *miR-18a*, *miR-145*, *miR-99a*	*miR-17*	Blood cells	[23]
*miR-301a-3p*, *miR-424-5p*, *miR-27a-3p*	*miR-205-5p*	Plasma	[17,24]
*miR-193b-3p*, *miR-720*, *miR-205-5p*, *miR-126-5p*, *miR-211-5p*, *miR-206*, *miR-550a-3p*, *miR-627-5p*, *miR-629-5p*	*miR-204-5p*, *miR-182-5p*, *miR-301a-3p*, *miR-200c-3p*, *miR-28-5p*, *miR-27a-3p*, *miR-197-3p*, *miR-374a-5p*	Serum	[25]
*miR-15b-5p*, *miR-149-3p*, *miR-150-5p*, *miR-155-5p*	*miR-193a-3p*, *miR-524-5p*	Plasma	[17,26]
-	*miR-29c-5p*, *miR-324-3p*	Serum	[27]
-	*miR-125b*	Serum and exosomes	[17,28]
*miR-20a*, *miR of the 17–92 complex*, *miR-125b*, *miR-146a*, *miR-155*, *miR-181a*, *miR-223*	-	Plasma	[29]
*miR-18a-5p*, *miR-146a-5p*, *miR-363-3p*	-	Melanoma tissue	[30]
*miR-122-5p*	-	Melanoma tissue and melanocytic nevi tissue	[31]
*miR-31-5p*, *miR-21-5p*	*miR-211-5p*, *miR-125a-5p*, *miR-125b-5p*, *miR-100 5p*	Melanoma tissue and melanocytic nevi tissue	[32]

**Table 2:** Diagnostic biomarkers of circulating microRNAs for melanoma. Metastatic melanoma patients compared with non-metastatic melanoma patients and/or patients with up-or down-regulation correlating with a poor prognosis of the disease.

Overexpressing microRNA-Candidates	Down-Expressing microRNA-Candidates	Sample, Tissue	Source Link
*miR-193b-3p*, *miR-720*	-	Serum	[25]
*miR-199a-5p*, *miR-150*, *miR-424*	*miR-15b*, *miR-33a*	Serum	[33]
-	*miR-200c-3p*	Plasma	[26]
-	*miR-16*	Serum	[34]
-	*miR-206*	Serum	[35]
*miR-21*	-	Plasma	[36]
*miR-221*	-	Serum	[37]
*miR-210*	-	Plasma	[38]
*miR-150*, *miR-30d*, *miR-15b*, *miR-425*	-	Serum	[39]

**Table 3:** Nucleotide sequences of candidate diagnostic microRNAs.

microRNAs	Sequence
*hsa-miR-155-5p*	UUAAUGCUAAUCGUGAUAGGGGUU
*hsa-miR-149-3p*	AGGGAGGGACGGGGCUGUGC
*hsa-miR-150-5p*	UCUCCCAACCCUUGUACCAGUG
*hsa-miR-193a-3p*	AACUGGCCUACAAAGUCCCAGU
*hsa-miR-21-5p*	AUGCUUAUCAGACUGAUGUUGA

**Table 4:** Characteristics of melanoma patients and healthy donors.

№	Gender	Age	Diagnosis	Stage	Patient Groups
1	male	69	Cutaneous melanoma	IV	Melanoma patients’ group
2	female	42	Nodular melanoma with epithelioid cells	IV	
3	female	37	Cutaneous melanoma	IV	
4	female	71	Cutaneous melanoma	IV	
5	male	68	Melanoma of anterior abdominal wall	IV	
6	female	45	Malignant melanoma of left lower limb	IV	
7	female	68	Malignant melanoma of left lower limb	IV	
8	male	67	Cutaneous melanoma	IV	
9	male	48	Cutaneous melanoma	IV	
10	male	41	Melanoma of anterior abdominal wall	IV	
11	female	52	Cutaneous melanoma	IV	
12	female	50	Cutaneous melanoma	IV	
13	male	69	Healthy	-	Healthy donors’ group
14	male	59	Healthy	-	
15	female	58	Healthy	-	
16	female	42	Healthy	-	
17	male	48	Healthy	-	
18	female	39	Healthy	-	
19	female	39	Healthy	-	
20	male	55	Healthy	-	
21	female	48	Healthy	-	
22	female	67	Healthy	-	
23	male	71	Healthy	-	
24	female	45	Healthy	-	

## Data Availability

The data presented in this study are available on request from the corresponding author.

## Supplementary Materials

The following supporting information can be downloaded at: https://www.mdpi.com/article/10.3390/ijms24119160/s1.

## References

[1] Erdei E., Torres S.M. (2010). A new understanding in the epidemiology of melanoma. Expert Rev. Anticancer. Ther..

[2] Schadendorf D., van Akkooi A.C.J., Berking C., Griewank K.G., Gutzmer R., Hauschild A., Stang A., Roesch A., Ugurel S. (2018). Melanoma. Lancet.

[3] Rigel D.S., Carucci J.A. (2000). Malignant melanoma: Prevention, early detection, and treatment in the 21st century. CA A Cancer J. Clin..

[4] Siegel R.L., Miller K.D., Jemal A. (2017). Cancer Statistics, 2017. CA Cancer J. Clin..

[5] Crocetti E., Mallone S., Robsahm T.E., Gavin A., Agius D., Ardanaz E., Lopez M.-D.C., Innos K., Minicozzi P., Borgognoni L. (2015). Survival of patients with skin melanoma in Europe increases further: Results of the EUROCARE-5 study. Eur. J. Cancer.

[6] Houghton A.N., Polsky D. (2002). Focus on melanoma. Cancer Cell.

[7] Liu Y., Sheikh M.S. (2014). Melanoma: Molecular Pathogenesis and Therapeutic Management. Mol. Cell. Pharmacol..

[8] O’Brien J., Hayder H., Zayed Y., Peng C. (2018). Overview of MicroRNA Biogenesis, Mechanisms of Actions, and Circulation. Front. Endocrinol..

[9] Gellrich F.F., Schmitz M., Beissert S., Meier F. (2020). Anti-PD-1 and Novel Combinations in the Treatment of Melanoma—An Update. J. Clin. Med..

[10] Calin G.A., Dumitru C.D., Shimizu M., Bichi R., Zupo S., Noch E., Aldler H., Rattan S., Keating M., Rai K. (2002). Frequent deletions and down-regulation of micro- RNA genes *miR15* and *miR16* at 13q14 in chronic lymphocytic leukemia. Proc. Natl. Acad. Sci. USA.

[11] Cheng L., Lopez-Beltran A., Massari F., MacLennan G.T., Montironi R. (2018). Molecular testing for BRAF mutations to inform melanoma treatment decisions: A move toward precision medicine. Mod. Pathol..

[12] Sidorova E.A., Zhernov Y.V., Antsupova M.A., Khadzhieva K.R., Izmailova A.A., Kraskevich D.A., Belova E.V., Simanovsky A.A., Shcherbakov D.V., Zabroda N.N. (2023). The Role of Different Types of microRNA in the Pathogenesis of Breast and Prostate Cancer. Int. J. Mol. Sci..

[13] Mitchell P.S., Parkin R.K., Kroh E.M., Fritz B.R., Wyman S.K., Pogosova-Agadjanyan E.L., Peterson A., Noteboom J., O’Briant K.C., Allen A. (2008). Circulating microRNAs as stable blood-based markers for cancer detection. Proc. Natl. Acad. Sci. USA.

[14] Chen X., Ba Y., Ma L., Cai X., Yin Y., Wang K., Guo J., Zhang Y., Chen J., Guo X. (2008). Characterization of microRNAs in serum: A novel class of biomarkers for diagnosis of cancer and other diseases. Cell. Res..

[15] Bradish J.R., Cheng L. (2014). Molecular pathology of malignant melanoma: Changing the clinical practice paradigm toward a personalized approach. Hum. Pathol..

[16] Felicetti F., Errico M.C., Bottero L., Segnalini P., Stoppacciaro A., Biffoni M., Felli N., Mattia G., Petrini M., Colombo M.P. (2008). The Promyelocytic Leukemia Zinc Finger–MicroRNA-221/-222 Pathway Controls Melanoma Progression through Multiple Oncogenic Mechanisms. Cancer Res..

[17] Kanemaru H., Fukushima S., Yamashita J., Honda N., Oyama R., Kakimoto A., Masuguchi S., Ishihara T., Inoue Y., Jinnin M. (2011). The circulating microRNA-221 level in patients with malignant melanoma as a new tumor marker. J. Dermatol. Sci..

[18] Qian L.-Y., Li P., He Q.-Y., Luo C.-Q. (2014). Circulating miR-221 Expression Level and Prognosis of Cutaneous Malignant Melanoma. Experiment.

[19] Flaherty K.T., Infante J.R., Daud A., Gonzalez R., Kefford R.F., Sosman J., Hamid O., Schuchter L., Cebon J., Ibrahim N. (2012). Combined BRAF and MEK Inhibition in Melanoma with BRAF V600 Mutations. N. Engl. J. Med..

[20] Varrone F., Caputo E. (2020). The miRNAs Role in Melanoma and in Its Resistance to Therapy. Int. J. Mol. Sci..

[21] Li L.N., Zhang H.D., Zhi R., Uuan S.J. (2011). Down-regulation of some miRNAs by degradaing their precorses contributes to anti-cancer effect of mistletoe lectin-I. Br. J. Pharmacok..

[22] Váraljai R., Elouali S., Lueong S., Wistuba-Hamprecht K., Seremet T., Siveke J., Becker J., Sucker A., Paschen A., Horn P. (2021). The predictive and prognostic significance of cell-free DNA concentration in melanoma. J. Eur. Acad. Dermatol. Venereol..

[23] Margue C., Reinsbach S., Philippidou D., Beaume N., Walters C., Schneider J.G., Nashan D., Behrmann I., Kreis S. (2015). Comparison of a healthy miRNome with melanoma patient miRNomes: Are microRNAs suitable serum biomarkers for cancer?. Oncotarget.

[24] Mumford S.L., Towler B.P., Pashler A.L., Gilleard O., Martin Y., Newbury S.F. (2018). Circulating MicroRNA Biomarkers in Melanoma: Tools and Challenges in Personalised Medicine. Biomolecules.

[25] Stark M.S., Klein K., Weide B., Haydu L.E., Pflugfelder A., Tang Y.H., Palmer J.M., Whiteman D.C., Scolyer R.A., Mann G.J. (2015). The Prognostic and Predictive Value of Melanoma-related MicroRNAs Using Tissue and Serum: A MicroRNA Expression Analysis. Ebiomedicine.

[26] Achberger S., Aldrich W., Tubbs R., Crabb J.W., Singh A.D., Triozzi P.L. (2014). Circulating immune cell and microRNA in patients with uveal melanoma developing metastatic disease. Mol. Immunol..

[27] Friedman E.B., Shang S., de Miera E.V.-S., Fog J.U., Teilum M.W., Ma M.W., Berman R.S., Shapiro R.L., Pavlick A.C., Hernando E. (2012). Serum microRNAs as biomarkers for recurrence in melanoma. J. Transl. Med..

[28] Tian R., Liu T., Qiao L., Gao M., Li J. (2015). Decreased serum microRNA-206 level predicts unfavorable prognosis in patients with melanoma. Int. J. Clin. Exp. Pathol..

[29] Greenberg E., Besser M.J., Ben-Ami E., Shapira-Frommer R., Itzhaki O., Zikich D., Levy D., Kubi A., Eyal E., Onn A. (2013). A comparative analysis of total serum miRNA profiles identifies novel signature that is highly indicative of metastatic melanoma: A pilot study. Biomarkers.

[30] Aksenenko M., Palkina N., Komina A., Tashireva L., Ruksha T. (2019). Differences in microRNA expression between melanoma and healthy adjacent skin. BMC Dermatol..

[31] Leidinger P., Keller A., Borries A., Reichrath J., Rass K., Jager S.U., Lenhof H.-P., Meese E. (2010). High-throughput miRNA profiling of human melanoma blood samples. BMC Cancer.

[32] Torres R., Lang U.E., Hejna M., Shelton S.J., Joseph N.M., Shain A.H., Yeh I., Wei M.L., Oldham M.C., Bastian B.C. (2020). MicroRNA Ratios Distinguish Melanomas from Nevi. J. Investig. Dermatol..

[33] Saldanha G., Potter L., Shendge P., Osborne J., Nicholson S., Yii N., Varma S., Aslam M.I., Elshaw S., Papadogeorgakis E. (2013). Plasma MicroRNA-21 Is Associated with Tumor Burden in Cutaneous Melanoma. J. Investig. Dermatol..

[34] Tan G.W., Khoo A.S.B., Tan L.P. (2015). Evaluation of extraction kits and RT-qPCR systems adapted to high-throughput platform for circulating miRNAs. Sci. Rep..

[35] van Laar R., Lincoln M., Van Laar B. (2018). Development and validation of a plasma-based melanoma biomarker suitable for clinical use. Br. J. Cancer.

[36] Fogli S., Polini B., Carpi S., Pardini B., Naccarati A., Dubbini N., Lanza M., Breschi M.C., Romanini A., Nieri P. (2017). Identification of plasma microRNAs as new potential biomarkers with high diagnostic power in human cutaneous melanoma. Tumor Biol..

[37] Li J., Zhao R., Fang R., Wang J. (2018). miR-122-5p inhibits the proliferation of melanoma cells by targeting NOP14. Nan Fang Yi Ke Da Xue Xue Bao = J. South. Med. Univ..

[38] Alegre E., Sanmamed M.F., Rodriguez C., Carranza O., Martín-Algarra S., González A. (2014). Study of Circulating MicroRNA-125b Levels in Serum Exosomes in Advanced Melanoma. Arch. Pathol. Lab. Med..

[39] Fleming N.H., Zhong J., da Silva I.P., de Miera E.V.-S., Brady B., Han S.W., Hanniford D., Wang J., Shapiro R.L., Hernando E. (2015). Serum-based miRNAs in the prediction and detection of recurrence in melanoma patients. Cancer.

[40] Farazi T.A., Hoell J.I., Morozov P., Tuschl T. (2013). MicroRNAs in human cancer. Adv. Exp. Med. Biol..

[41] Polini B., Carpi S., Doccini S., Citi V., Martelli A., Feola S., Santorelli F.M., Cerullo V., Romanini A., Nieri P. (2020). Tumor Suppressor Role of hsa-miR-193a-3p and -5p in Cutaneous Melanoma. Int. J. Mol. Sci..

[42] Yong F.L., Law C.W., Wang C.W. (2013). Potentiality of a triple microRNA classifier: miR-193a-3p, miR-23a and miR-338-5p for early detection of colorectal cancer. BMC Cancer.

[43] Hu T., Chong Y., Cai B., Liu Y., Lu S., Cowell J.K. (2019). DNA methyltransferase 1–mediated CpG methylation of the miR-150-5p promoter contributes to fibroblast growth factor receptor 1–driven leukemogenesis. J. Biol. Chem..

[44] Lin Y.-C., Kuo M.-W., Yu J., Kuo H.-H., Lin R.-J., Lo W.-L., Yu A. (2008). c-Myb Is an Evolutionary Conserved miR-150 Target and miR-150/c-Myb Interaction Is Important for Embryonic Development. Mol. Biol. Evol..

[45] Leone E., Morelli E., Di Martino M.T., Amodio N., Foresta U., Gullà A., Rossi M., Neri A., Giordano A., Munshi N.C. (2013). Targeting miR-21 Inhibits In Vitro and In Vivo Multiple Myeloma Cell Growth. Clin. Cancer Res..

[46] Bovell L.C., Shanmugam C., Putcha B.-D.K., Katkoori V.R., Zhang B., Bae S., Singh K.P., Grizzle W.E., Manne U. (2013). The Prognostic Value of MicroRNAs Varies with Patient Race/Ethnicity and Stage of Colorectal Cancer. Clin. Cancer Res..

[47] del Campo S.E.M., Latchana N., Levine K.M., Grignol V.P., Fairchild E.T., Jaime-Ramirez A.C., Dao T.-V., Karpa V.I., Carson M., Ganju A. (2015). MiR-21 Enhances Melanoma Invasiveness via Inhibition of Tissue Inhibitor of Metalloproteinases 3 Expression: In Vivo Effects of MiR-21 Inhibitor. PLoS ONE.

[48] Xu L.-F., Wu Z.-P., Chen Y., Zhu Q.-S., Hamidi S., Navab R. (2014). MicroRNA-21 (miR-21) Regulates Cellular Proliferation, Invasion, Migration, and Apoptosis by Targeting PTEN, RECK and Bcl-2 in Lung Squamous Carcinoma, Gejiu City, China. PLoS ONE.

[49] Babu A., Ramesh R. (2017). Multifaceted Applications of Chitosan in Cancer Drug Delivery and Therapy. Mar Drugs.

[50] Vznuzdaeva O.A., Zverev G.A., Molodtsov I.V. (1984). Effect of chitosan on IgM and IgG antibody-producing cells in mice. Immunologiya.

[51] Azuma K., Osaki T., Minami S., Okamoto Y. (2015). Anticancer and Anti-Inflammatory Properties of Chitin and Chitosan Oligosaccharides. J. Funct. Biomater..

[52] Huang R., Mendis E., Rajapakse N., Kim S.-K. (2006). Strong electronic charge as an important factor for anticancer activity of chitooligosaccharides (COS). Life Sci..

[53] Xu W., Jiang C., Kong X., Liang Y., Rong M., Liu W. (2012). Chitooligosaccharides and N-acetyl-D-glucosamine stimulate peripheral blood mononuclear cell-mediated antitumor immune responses. Mol. Med. Rep..

[54] Zou P., Yang X., Zhang Y., Du P., Yuan S., Yang D., Wang J. (2016). Antitumor Effects of Orally and Intraperitoneally Administered Chitosan Oligosaccharides (COSs) on S180-Bearing/Residual Mouse. J. Food Sci..

[55] Srinivasan H., Kanayairam V., Ravichandran R. (2018). Chitin and chitosan preparation from shrimp shells Penaeus monodon and its human ovarian cancer cell line, PA-1. Int. J. Biol. Macromol..

[56] Julious S.A. (2005). Sample size of 12 per group rule of thumb for a pilot study. Pharm. Stat..

[57] Mikhaĭlova I.N., Lukashina M.I., Baryshnikov AIu Morozova L.F., Burova O.S., Palkina T.N., Kozlov A.M., Golubeva V.A., Cheremushkin E.A., Doroshenko M.B., Georgiev G.P. (2005). Melanoma cell lines as the basis for antitumor vaccine preparation. Vestn. Ross. Akad. Meditsinskikh Nauk..

[58] Dobosz P., Dzieciątkowski T. (2019). The Intriguing History of Cancer Immunotherapy. Front. Immunol..

[59] Troy E., Tilbury M.A., Power A.M., Wall J.G. (2021). Nature-Based Biomaterials and Their Application in Biomedicine. Polymers.

[60] Habtemariam S. (2020). Trametes versicolor (Synn. Coriolus versicolor) Polysaccharides in Cancer Therapy: Targets and Efficacy. Biomedicines.

[61] Harhaji L., Mijatović S., Maksimović-Ivanić D., Stojanović I., Momčilović M., Maksimović V., Tufegdžić S., Marjanović Ž., Mostarica-Stojković M., Vučinić Ž. (2008). Anti-tumor effect of Coriolus versicolor methanol extract against mouse B16 melanoma cells: In vitro and in vivo study. Food Chem. Toxicol..

[62] Stevenson F.J. (1994). Humus Chemistry: Genesis, Composition, Reactions.

[63] Directory of Medicines. http://www.rlsnet.ru/mnn_index_id_2851.htm.

[64] Zhernov Y.V., Kremb S., Helfer M., Schindler M., Harir M., Mueller C., Hertkorn N., Avvakumova N.P., Konstantinov A.I., Brack-Werner R. (2016). Supramolecular combinations of humic polyanions as potent microbicides with polymodal anti-HIV-activities. New. J. Chem..

[65] Badun G.A., Chernysheva M.G., Zhernov Y.V., Poroshina A.S., Smirnov V.V., Pigarev S.E., Mikhnevich T.A., Volkov D.S., Perminova I.V., Fedoros E.I. (2021). A Use of Tritium-Labeled Peat Fulvic Acids and Polyphenolic Derivatives for Designing Pharmacokinetic Experiments on Mice. Biomedicines.

[66] Fedoros E.I., Orlov A.A., Zherebker A., Gubareva E.A., Maydin M.A., Konstantinov A.I., Krasnov K.A., Karapetian R.N., Izotova E.I., Pigarev S.E. (2018). Novel water-soluble lignin derivative BP-Cx-1: Identification of components and screening of potential targets in silico and in vitro. Oncotarget.

[67] Zhernov Y.V., Konstantinov A.I., Zherebker A., Nikolaev E., Orlov A., Savinykh M.I., Kornilaeva G.V., Karamov E.V., Perminova I.V. (2021). Antiviral activity of natural humic substances and shilajit materials against HIV-1: Relation to structure. Environ. Res..

[68] Avvakumova N., Kamilov F., Zhdanova A., Men’shikova I., Zhernov Y., Krivopalova M., Glubokova M., Katunina E. (2018). The influence of humus acids of peloids and its components on free radical processes. Biomeditsinskaya Khimiya.

[69] Orlov A.A., Zherebker A., Eletskaya A.A., Chernikov V.S., Kozlovskaya L.I., Zhernov Y.V., Kostyukevich Y., Palyulin V.A., Nikolaev E.N., Osolodkin D.I. (2019). Examination of molecular space and feasible structures of bioactive components of humic substances by FTICR MS data mining in ChEMBL database. Sci. Rep..

[70] Botes M.E., Dekker J., van Rensburg C.E.J. (2002). Phase I Trial with Oral Oxihumate in HIV-Infected Patients. Drug Dev. Res..

[71] Jooné G.K., Dekker J., van Rensburg C.E.J. (2003). Investigation of the Immunostimulatory Properties of Oxihumate. Z. Naturforsch. C.

[72] Sanmiguel P.R., Rondón B.I. (2016). Supplementation with humic substances affects the innate immunity in layer hens in posfasting phase. Rev. MVZ Córdoba.

[73] Vasnev V.A., Tarasov A.I., Markova G.D., Vinogradova S.V., Garkusha O.G. (2006). Synthesis and properties of acylated chitin and chitosan derivatives. Carbohydr. Polym..

[74] Andersen C.L., Jensen J.L., Ørntoft T.F. (2004). Normalization of Real-Time Quantitative Reverse Transcription-PCR Data: A Model-Based Variance Estimation Approach to Identify Genes Suited for Normalization, Applied to Bladder and Colon Cancer Data Sets. Cancer Res..

