# Supplementary material for: Determination of Common microRNA Biomarker Candidates in Stage IV Melanoma Patients and a Human Melanoma Cell Line: A Potential Anti-Melanoma Agent Screening Model
Source: Int J Mol Sci. 2023 May 23;24(11):9160. doi: 10.3390/ijms24119160 (PMC10252288; doi:10.3390/ijms24119160)
Supplement: Supplementary file 1 [file ijms-24-09160-s001.zip › ijms-2352423-Supplementary File S1.pdf]

*Supplementary Materials*

# Determination of Common microRNA Biomarker Candidates in Stage IV Melanoma Patients and a Human Melanoma Cell Line: A Potential Anti-Melanoma Agent Screening Model


**Elena Antonova [1], Anastasia Hambikova [1], Denis Shcherbakov [2], Vitaly Sukhov [2], Sonya Vysochanskaya [2], Inna Fadeeva [3], Denis Gorshenin [4], Ekaterina Sidorova [2], Maria Kashutina [5,6,7], Alina Zhdanova [8], Oleg Mitrokhin [2], Nadezhda Avvakumova [8] and Yury Zhernov [2,9,10,11,*]**

1 Research Center for Fundamental and Applied Problems of Bioecology and Biotechnology, I.N. Ulyanov Ulyanovsk State Pedagogical University, 432700 Ulyanovsk, Russia
2 Department of General Hygiene, F. Erismann Institute of Public Health, I.M. Sechenov First Moscow State Medical University (Sechenov University), 119435 Moscow, Russia
3 Department of English Language, Institute of World Economy, Diplomatic Academy of the Russian Foreign Ministry, 119992 Moscow, Russia
4 Laboratory of Innate Immunity, National Research Center—Institute of Immunology FMBA of Russia, 115522 Moscow, Russia
5 Loginov Moscow Clinical Scientific and Practical Center, 111123 Moscow, Russia
6 Department of Public Health Promotion, National Research Centre for Therapy and Preventive Medicine, 101990 Moscow, Russia
7 Department of Therapy, Clinical Pharmacology and Emergency Medicine, A.I. Yevdokimov Moscow State University of Medicine and Dentistry, 127473 Moscow, Russia
8 Department of Medical Chemistry, Samara State Medical University, 443099 Samara, Russia
9 Department of Chemistry, M.V. Lomonosov Moscow State University, 119991 Moscow, Russia
10 Center of Life Sciences, Skolkovo Institute of Science and Technology, 121205 Moscow, Russia
11 Center for Medical Anthropology, N.N. Miklukho-Maclay Institute of Ethnology and Anthropology of the Russian Academy of Sciences, 119017 Moscow, Russia
* Correspondence: zhernov@list.ru; Tel.: +7-9151-552-000


### *Obtaining fractions of humic substances from peloids*

The samples were obtained by subsequent extraction (fractionation) from low-mineralized silt sulfide muds (peloids) of the MRC "Sergievsky Mineral Waters" FMBA of Russia, the Sergievsky district of the Samara region, Russia. We obtained two fractions of humic substances: the alkaline-soluble fraction of humic acid (HA) and the alcohol-soluble fraction of hymatomelanic acid (HMA).

To destroy the organomineral complexes of peloids, eliminate sulfides and carbonates, native mud was treated with a 2M solution of hydrochloric acid without thermal exposure, which prevented the destruction of specific organic substances. The solvent was replaced daily until a negative reaction to ions was performed: the calcium cation–ammonium oxalate test, the iron (III) cation–potassium thiocyanate test, the carbonate anion–calcium chloride test, and the sulfide anion–copper (II) chloride test. After negative samples for ions, the precipitate was washed with hot water, followed by decantation and treatment on a filter with distilled water until a negative reaction for the chloride anion in the sample with silver nitrate.

In order to obtain a low-mineralized preparation, the extraction of specific organic substances was carried out with a 0.5 M sodium hydroxide solution in a peloid–solvent ratio of 1:10 no more than three times. In the course of this stage of the study, it was found that in subsequent portions, the number of mineral components increased significantly, which negatively affected the results of the experiments.

At this stage, a mixed fraction of HA and HMA was obtained. Next, the extract, which was a mixture of sodium salts HA and HMA, was converted into the H-form by passing through a KU-2 cation exchanger. The completeness of ion exchange was controlled by the constancy of the pH of the eluent solution on an EV-74 universal ion meter. The drug was brought to an air-dried state at a temperature of 35–40°C using forced ventilation.

For further fractionation of specific organic substances, a 50% sulfuric acid solution was added to pH 1.0. The acidity of the medium was determined by a universal indicator. The extract was left for 18–20 hours to achieve complete precipitation.

The supernatant liquid was decanted, filtered, and transferred to the adsorbed state on BAU-activated carbon, after which purification was carried out according to the Forsyth method. The residue on the filter was washed with water until a negative reaction to the sulfate anion with a solution of barium chloride, after which exhaustive extraction with ethanol was carried out until the extractant turned pale yellow. Thus, an HMA fraction was obtained. After distillation under vacuum of the maximum possible amount of the solvent, HMA was converted into the sodium form by dissolving in a solution of 0.1 M sodium hydroxide and precipitated by adding sulfuric acid to 1–2 pH units. The solution was left for 18–20 hours for complete precipitation. Reprecipitation was repeated twice to purify the preparation from mineral impurities. Then, the drug was converted into the H-form and dried at a temperature of 35–40°C using forced ventilation.

The filter residue after alcohol extraction is the HA fraction. HA was dissolved in a minimum amount of 0.02M sodium hydroxide solution, then precipitated with sulfuric acid. To obtain a low-ash preparation, reprecipitation was carried out twice, after which the alkaline solution was passed through a cation exchanger and dried.

Solid-phase extraction (SPE) followed by gradient elution on three Bond Elut cartridges—C8, C18 and CN-E—was used to obtain narrow fractions of HMA. The eluent was various mixtures of methanol and water in the ratios 100:0, 90:10 ... 10:90, 0:100. A total of 33 fractions were obtained, 11 from each of the 3 cartridges. Each SPE fraction was characterized by ion cyclotron resonance mass spectrometry (ICR-MS) data; cytotoxicity and anti-melanoma activity were measured.

UPLC fractionation of the eluate obtained after SPE extraction of the HMA sample was performed using an Acquity UPLC system (Waters) connected to a maXis Qq-TOF mass spectrometer (Bruker Daltonics, Bremen, Germany). Chromatographic separation was achieved on a Kinetex Phenyl PFP UPLC column (2.1 x 150 mm, 1.7 μm), Phenomenex. Gradient elution buffers were A ($H_2O$-MeOH: 95/5 containing 0.1% formic acid) and B (methanol) with a flow rate of 0.300 ml/min.

The column thermostat was set to 60°C and the sample manager temperature to 15°C. The injection volume was 10 μl and the HMA extract was administered four times. The spectra were obtained in continuous and centroid modes with a moving average of 2 and a scan rate of 1.0 Hz. Fractions were collected with a NanoMate fraction collector (TriVersa) in a 96-well plate. Fractions were freeze-dried and reconstituted in $ddH_20$ for testing anti-melanoma activity.

### *Obtaining chitosan fractions*

In our work, we used three fractions of chitosan with molecular weights of 10 kDa, 120 kDa, and 500 kDa, produced by Bioprogress Ltd., Shchyolkovo (Russia). The separation of samples and determination of molecular weight were performed by high-performance liquid chromatography with column calibration with reference dextrans. Three chitin fractions were obtained during the reaction of the above chitosans with acetic anhydride in the modification, as described in [73].

A portion of the chitosan fractions was completely dissolved in a 1% acetic acid solution to obtain a 0.05% solution. The polymer was precipitated by adding sodium hydroxide with a change in pH to 8–9. The resulting precipitate of hydrated chitosan was washed three times with distilled water and precipitated by centrifugation. After the third wash, a suspension of the polymer in phosphate-buffered saline (PBS) was obtained. The solution was sterilized by autoclaving.

To obtain a nanodispersion of the drug, a suspension was sounded twice on a high-power ultrasonic generator for 10 minutes under sterile conditions. The resulting suspension was passed through a glass fiber filter with a pore diameter of 1 μm. The drug concentration in the solution was determined by weighing the dry residue after freeze-drying.

All solutions were adjusted to a concentration of 5 mg/ml under sterile conditions. Working solutions were stored at 4°C for no more than 72 hours.
